# Neural Networks and Connectivity among Brain Regions

**DOI:** 10.3390/brainsci12030346

**Published:** 2022-03-03

**Authors:** Mauro Ursino, Elisa Magosso, Manuela Petti

**Affiliations:** 1 Department of Electrical, Electronic and Information Engineering Guglielmo Marconi, Campus of Cesena, University of Bologna, Via Dell’Università 50, 47521 Cesena, Italy; elisa.magosso@unibo.it; 2 Department of Computer, Control and Management Engineering, Sapienza University of Rome, Via Ariosto, 25, 00185 Roma, Italy; manuela.petti@uniroma1.it

As is widely understood, brain functioning depends on the interaction among several neural populations, which are linked via complex connectivity circuits and work together (in antagonistic or synergistic ways) to exchange information, synchronize their activity, adapt plastically to external stimuli or internal requirements, and more generally to participate in solving multifaceted cognitive tasks. Indeed, from the very beginning of neuroscience, questions regarding how different regions participate in cognition and their reciprocal roles have attracted the interest of researchers. Thanks to the development of advanced techniques for functional brain imaging and the tuning of sophisticated signal-processing methods, the interest in brain connectivity estimation has experienced an enormous expansion.

All papers in this Special Issue are focused on connectivity, which is the influence that the activity in one brain region can have on that in another brain region. However, this apparently simple definition encompasses a series of problems that warrant critical analysis. Although many papers have appeared recently, several aspects of brain connectivity are still controversial. The reader should be aware of the profound, important differences in the implications of this term, depending on the adopted methodologies and the context of the application. First, the definition of brain connectivity is not univocal, and diverse terms (structural, functional, and effective connectivity) are used in the literature, which are often confusing or ambiguous, depending on the approach used and on the aspects that should be emphasized. The crucial point here is that several different mathematical methods are employed in the literature to estimate connectivity from data—from straightforward statistical ones (such as correlations among time series) to more sophisticated methods using linear or non-linear models to derive causal links. The significance of the metrics obtained in this way is not equivalent. Hence, fundamental aspects in brain connectivity estimation and interpretation concern the reliability of the estimation techniques, their accuracy in different conditions, the neurophysiological significance of the connectivity values derived, and the kinds of neurological phenomena they can grasp.

A paper in this Special Issue (Ricci et al. [1]) is mainly focused on these aspects. In this work, several methods for the estimation of brain connectivity are compared using the signals generated through a neural mass model as ground truth, simulating several interconnected regions of interest. The results show that the capacity of the Granger causality to detect proper connectivity overcomes that of the other estimators (including transfer entropy, correlation, coherence, and phase synchronization). However, even more importantly, the results underline the strong effect that non-linearity can have on the estimated values. There is clear evidence that connectivity reflects the amount of information transmitted from one region to another, a quantity that can be vastly different from the strength of a causal link. Indeed, one area can have a strong causal impact on a second region (for instance, leading its activity to saturation without actual information exchange). This is an aspect that should be carefully taken into account when analyzing connectivity data, especially in conditions (such as task-dependent connectivity) characterized by a change in the working point (also see Ursino et al. [2]).

Furthermore, connectivity estimation methods differ significantly in relation to the data employed, particularly neuroimaging or neuroelectrical signals. As is widely understood, neuroimaging data allow a better spatial resolution and the study of deeper brain regions, such as the hippocampus or the amygdala, which are involved in fundamental cognitive tasks. However, they cannot analyze temporal aspects, such as brain waves, which play a pivotal role in modern cognitive neuroscience research.

In this Special Issue, several papers make use of functional neuroimaging data (fMRI) to derive connectivity: in particular, the papers by Chrobak et al. [3], De Asis-Cruz et al. [4], Khan et al. [5], and Liu et al. [6] are based on resting-state data, while only the paper by Tak et al. [7] makes use of fMRI during a motor task (right-hand movement) together with dynamic causal modeling.

On the other hand, the use of EEG data is receiving increasing attention in the current literature due to the simplicity of the experimental setup and its capacity to detect rapid temporal changes in neural activity. Three papers in this Special Issue infer connectivity from EEG ([8,9,10]). The first two papers use Granger connectivity (bivariate in Tarasi et al. [9] and multivariate in Formaggio et al. [8]). In contrast, the last paper (Taberna et al. [10]) uses a different approach based on band-limited power envelope correlations. It is worth noting that, in these three papers, data are acquired during resting-state activity, and all are based on source reconstruction in the cortex, although starting from different numbers of scalp electrodes (32, 64, and 256, respectively).

Of course, cortical source reconstruction from scalp EEG requires a model of the head. To solve this problem, several works in the literature employ template head models rather than head models of individual subjects. This limitation can become especially crucial when high-density scalp electrodes are used, since reconstruction inaccuracy can counteract the advantage of having many measurement sites. This problem is analyzed in the paper by Taberna et al. [10], who evaluate how the estimate of resting-state brain connectivity from high-density EEG data relies on creating an accurate head model. They conclude that the inaccuracies in head tissue segmentation have a higher impact on resting-state connectivity than inaccuracy in electrode positions.

While the previous findings were focused on the techniques adopted (kinds of signals, methods used to estimate connectivity, and causes of errors), further interesting concepts can be developed by considering the possible modern applications of connectivity studies. Indeed, only two papers focused on methodological aspects, i.e., the papers mentioned above by Ricci et al. [1] (based on simulated data and on the reliability of different connectivity estimators) and by Taberna et al. [10] (focusing on the accuracy of connectivity estimate vs. head modeling). In all the other papers, brain connectivity is used to investigate brain networks in clinical or subclinical subjects and compare them to healthy subjects (five papers) or to discriminate developmental or age aspects (two papers); in more general terms, all these papers try to find a discrimination criterion to differentiate classes of individuals. The basic assumption is that connectivity changes may contain important neuromarkers of brain disorders, even at subclinical levels, or of developmental states. Therefore, a connectivity analysis can provide substantial potential benefits for clinical practice in the near future.

Concerning clinical aspects, the paper by Chrobak et al. [3] compares brain activity in patients with *euthymic bipolar disorder* (BD) and healthy controls (HC) using resting-state fMRI and exploits functional connectivity based on the amplitude of low-frequency fluctuations, together with regional homogeneity. The study reveals differences in the spatial patterns of brain activity between euthymic BD patients and HC and the existence of a significant hyper-connectivity between several brain regions within the BD group.

Liu et al. [6] analyzed subjects with *mild dementia* to understand the possible beneficial effects of cognitive stimulation therapy (a kind of therapy based on non-pharmacological interventions) on cognitive and brain mechanisms. To this end, they collected structural MRI and resting-state functional MRI data and evaluated connectivity through correlation and anticorrelation analysis. An interesting result is that functional connectivity in the default mode network, which has implications in episodic memory and self-representation, improves after the therapy.

Two papers in this Special Issue deal with *autistic spectrum disorder* (ASD). The paper by Khan et al. [5] proposes a deep learning-based feature selection approach for the classification of ASD. The method employs three stages, in which neural networks are trained on the correlation-based connectivity matrix, and finally a subset of features is extracted, which is able to discriminate between the autistic patients and healthy controls. Conversely, the paper by Tarasi et al. [9] uses both temporal and spectral Granger connectivity networks, based on source electrical activity reconstructed from resting-state EEG data, to infer differences in connectivity between two classes of non-clinical subjects with lower or higher autistic traits. The fundamental result is that the brain network in individuals with higher autistic traits is more based on bottom-up information. This result seems to replicate those found in some clinical forms of autism.

The paper by Formaggio et al. [8] is also based on source activity reconstruction from resting-state EEG data and on the estimation of Granger connectivity networks. The aim is to discover differences in connectivity patterns between subjects with *Parkinson’s disease* (PD) and healthy controls. In this work, as in the previous, some indices from graph theory are used to summarize the results. The main conclusion is that motor brain areas in the PD network appear more isolated compared with the healthy network, mainly due to a reduced information transmission from parietal and occipital regions. These changes may reflect an impoverishment in sensory–motor abilities in this pathological state.

The last two papers investigate temporal changes in functional connectivity. In particular, the study by De Asis Cruz et al. [4] analyzes how resting-state functional connectivity, derived from fMRI, can vary in healthy fetuses between 19 and 40 gestational weeks. A fundamental turning point seems to occur at 30–31 weeks, characterized by a shift from a connectivity network that is more based on endogenous neural activity, to a network that is more based on sensory-driven inputs. Finally, Tak et al. [7], using task fMRI data, analyzed differences in connectivity in the motor cortex as a function of age (using an extensive data set of participants aged 18 to 88). This is the only study in this Special Issue based on a motor task and structural equation modeling. The results emphasize the importance of age changes in inter-hemispheric connectivity: older people are characterized by an increase in connections from the contralateral supplementary motor cortex and from the dorsal premotor cortex to the ipsilateral primary motor area and by a decrease in the connections from the contralateral primary to the ipsilateral primary motor area.

In summary, what are the main conclusions that we can draw from reading papers in this Special Issue or, more generally, from connectivity studies in the recent literature? First, the significance of connectivity metrics, their accuracy and the kind of information they can collect still require further assessment. As pointed out by the recent work of Reid et al. [11], many ambiguities still need to be overcome to achieve a full mechanistic explanation of neural network processes. In this regard, methods for the validation of connectivity metrics are still necessary and may play a pivotal role in future years, for instance, based on simulation models or empirical data. More generally, methodological studies are still critical and deserve particular attention to avoid misleading information being used in the construction of theoretical neuroscience.

Second, large interconnected networks, such as those emerging from functional neuroimaging or EEG data, are difficult to summarize into a simple theoretical structure with a limited number of relevant features. In this regard, increasing attention is paid to measures extracted from graph theory, which can be helpful to reach a clear understanding of brain network organization and of the main changes that can occur in network structural properties. Furthermore, recent artificial intelligence techniques (particularly deep learning) applied to connectivity matrices have achieved promising results thanks to their ability to extract latent relevant lower-dimension features from high-dimensional input data. The latter can be used not only to improve classification performances, e.g., between pathological and healthy subjects, but if interpreted via appropriate interpretation techniques (an issue still largely unexplored), could provide information about underlying neuroanatomical or neurofunctional alterations [12].

Third, as many papers in this issue demonstrate, connectivity studies are now frequently used to derive information on clinical conditions (e.g., Parkinson’s disease, dementia, autism spectrum disorder, and bipolar disorder) or to assess temporal changes in the brain. These studies have a double function: to gain a deeper insight into the mechanisms of brain function in healthy conditions and pathological states and, consequently, to drive future innovative therapeutic interventions. Indeed, if we know which portions of a neural circuitry are altered in neurological disease and which brain regions play a major role, these can become the target of therapies aiming to recover a normal condition or, at least, to attenuate the most severe symptoms. In this regard, a particularly interesting aspect that still warrants future investigation is that brain connectivity may provide highly sensitive neuromarkers of brain disorders, which are able to catch even subtle modifications due to disorder progression or improvement and capture alterations even at an early pre-clinical stage. Thus, connectivity-based neuromarkers could also prove valuable for predicting the clinical evolution of a disorder or the behavioral/motor outcome of a therapeutic intervention.

## References

[1] Ricci G., Magosso E., Ursino M. (2021). The Relationship between Oscillations in Brain Regions and Functional Connectivity: A Critical Analysis with the Aid of Neural Mass Models. Brain Sci..

[2] Ursino M., Ricci G., Astolfi L., Pichiorri F., Petti M., Magosso E. (2021). A Novel Method to Assess Motor Cortex Connectivity and Event Related Desynchronization Based on Mass Models. Brain Sci..

[3] Chrobak A.A., Bohaterewicz B., Sobczak A.M., Marszał-Wiśniewska M., Tereszko A., Krupa A., Ceglarek A., Fafrowicz M., Bryll A., Marek T. (2021). Time-Frequency Characterization of Resting Brain in Bipolar Disorder during Euthymia—A Preliminary Study. Brain Sci..

[4] De Asis-Cruz J., Barnett S.D., Kim J.-H., Limperopoulos C. (2021). Functional Connectivity-Derived Optimal Gestational-Age Cut Points for Fetal Brain Network Maturity. Brain Sci..

[5] Khan N.A., Waheeb S.A., Riaz A., Shang X. (2020). A Three-Stage Teacher, Student Neural Networks and Sequential Feed Forward Selection-Based Feature Selection Approach for the Classification of Autism Spectrum Disorder. Brain Sci..

[6] Liu T., Spector A., Mograbi D.C., Cheung G., Wong G.H.Y. (2021). Changes in Default Mode Network Connectivity in Resting-State FMRI in People with Mild Dementia Receiving Cognitive Stimulation Therapy. Brain Sci..

[7] Tak Y.W., Knights E., Henson R., Zeidman P. (2021). Ageing and the Ipsilateral M1 BOLD Response: A Connectivity Study. Brain Sci..

[8] Formaggio E., Rubega M., Rupil J., Antonini A., Masiero S., Toffolo G.M., Del Felice A. (2021). Reduced Effective Connectivity in the Motor Cortex in Parkinson’s Disease. Brain Sci..

[9] Tarasi L., Magosso E., Ricci G., Ursino M., Romei V. (2021). The Directionality of Fronto-Posterior Brain Connectivity Is Associated with the Degree of Individual Autistic Traits. Brain Sci..

[10] Taberna G.A., Samogin J., Marino M., Mantini D. (2021). Detection of Resting-State Functional Connectivity from High-Density Electroencephalography Data: Impact of Head Modeling Strategies. Brain Sci..

[11] Reid A.T., Headley D.B., Mill R.D., Sanchez-Romero R., Uddin L.Q., Marinazzo D., Lurie D.J., Valdés-Sosa P.A., Hanson S.J., Biswal B.B. (2019). Advancing Functional Connectivity Research from Association to Causation. Nat. Neurosci..

[12] Du Y., Fu Z., Calhoun V.D. (2018). Classification and Prediction of Brain Disorders Using Functional Connectivity: Promising but Challenging. Front. Neurosci..

